# 2358. Safety Profiles of Three Major Types of COVID-19 Vaccine among Two Cohorts of People Living with HIV

**DOI:** 10.1093/ofid/ofad500.1979

**Published:** 2023-11-27

**Authors:** Thana Khawcharoenporn, Sirashat Hanvivattanakul

**Affiliations:** Thammasat University, Bangkok, Krung Thep, Thailand; Faculty of Medicine, Thammasat University, Pathumthani, Pathum Thani, Thailand

## Abstract

**Background:**

Existing data on adverse effects (AEs) of COVID-19 vaccines among people living with human immunodeficiency virus (PLHIV) are currently limited.

**Methods:**

A cohort study was conducted among PLHIV receiving care at two clinics during COVID-19 epidemics in Thailand (2021-2022). The PLHIV received various types and regimens of COVID-19 vaccines based on vaccine availability and the national policies. Data on vaccine types and AEs were collected and assessed using the online survey system.

**Results:**

Of the 398 vaccinated PLHIV, 365 (92%) had CD4 count at enrollment ≥ 200 cells/µL, 382 (96%) were virologic suppressed, 153 received two doses and 245 received three doses of COVID-19 vaccines. Inactivated and viral vector were the most common vaccine types received by the PLHIV as the first and second doses (47% and 56%, respectively), while mRNA vaccine was the most commonly used as a booster dose (57%). For the first and second vaccine doses, the most common AEs were fever (15% and 11%) and injection site pain (11% and 11%). The mRNA vaccine significantly caused more overall AEs, injection pain, fatigue, and rashes than the other two types, while the mRNA and viral vector vaccines significantly caused more fever than the inactivated vaccine. For a booster dose, viral vector vaccine significantly caused more injection site pain and headache than the other two types. Most of the PLHIV reported that the AE intensity was similar between the first and second doses (49%) and was more for a booster dose compared to the first dose (50%). The majority of all AEs of the first, second and booster doses spontaneously recovered without treatment (55%, 59%, and 59%) or required symptomatic treatment without hospitalization (44%, 40% and 40%). By multivariable analysis, receipt of viral vector or mRNA vaccine and age less than 40 years were independently associated with any AEs of the primary series vaccines, while having AEs from the previous dose and female sex were factors associated with any AEs of a booster vaccine.

**Conclusion:**

The three types of COVID-19 vaccines were generally safe among PLHIV. Most of the AEs were non-severe and did not require hospitalization. PLHIV who were elderly, female or had AEs from the previous vaccine dose should be closely monitored for AEs of the vaccines.

**Disclosures:**

**All Authors**: No reported disclosures

